# SARS-CoV-2 vaccine effectiveness and clinical outcomes in hemodialysis patients: the NHIS-COVID-19 cohort study in South Korea

**DOI:** 10.3389/fpubh.2024.1372525

**Published:** 2024-05-09

**Authors:** Young-Ki Lee, Seon A. Jeong, Hayne Cho Park, Do Hyoung Kim, Kyung Don Yoo, Hye Eun Yoon, Yang Gyun Kim, Ajin Cho

**Affiliations:** ^1^Department of Internal Medicine, Kangnam Sacred Heart Hospital, Seoul, Republic of Korea; ^2^Hallym Kidney Research Institute, Hallym University College of Medicine, Seoul, Republic of Korea; ^3^Korean Society of Nephrology, Seoul, Republic of Korea; ^4^Basic-Clinical Convergence Research Institute, University of Ulsan, Dong-Gu, Ulsan, Republic of Korea; ^5^Department of Internal Medicine, Incheon St Mary's Hospital, College of Medicine, The Catholic University of Korea, Incheon, Republic of Korea; ^6^Division of Nephrology, Department of Internal Medicine, KyungHee University, Seoul, Republic of Korea

**Keywords:** COVID-19, SARS-CoV-2, vaccine, mortality, hemodialysis

## Abstract

**Background:**

Patients undergoing hemodialysis (HD) have a high risk of novel coronavirus disease 2019 (COVID-19) and poor clinical outcomes. This study aimed to investigate severe acute respiratory syndrome coronavirus 2 (SARS-CoV-2) vaccine effectiveness against infection and deaths in the South Korean population undergoing HD.

**Methods:**

We conducted a retrospective cohort study to compare the incidence of COVID-19 and post-diagnosis mortality between patients who were either never vaccinated or fully or partially vaccinated. The Korean nationwide COVID-19 registry and the Korean National Health Insurance Service databases were used. Adult patients without a history of COVID-19 were included between October 8, 2020, and December 31, 2021. The study outcomes were COVID-19 diagnosis, severe clinical COVID-19-related events, and post-diagnosis death.

**Results:**

Eighty-five thousand eighteen patients undergoing HD were included, of whom 69,601 were fully vaccinated, 2,213 were partially vaccinated and 13,204 were unvaccinated. Compared with the unvaccinated group, the risk of being diagnosed with COVID-19 in patients who were fully vaccinated decreased during the study period (adjusted odds ratio [aOR] = 0.147; 95% confidence interval [CI] = 0.135–0.159). There were 1,140 (1.3%) patients diagnosed with COVID-19. After diagnosis, fully vaccinated patients were significantly less likely to die than unvaccinated patients (aOR = 0.940; 95% CI = 0.901–0.980) and to experience severe clinical events (aOR = 0.952; 95% CI = 0.916–0.988).

**Conclusion:**

Full vaccination against COVID-19 was associated with a reduced risk of both infection and severe clinical outcomes in the South Korean population undergoing HD. These findings support the use of vaccination against SARS-CoV-2 among patients undergoing HD.

## Introduction

Since the World Health Organization characterized as a pandemic the coronavirus disease 2019 (COVID-19), which is caused by the severe acute respiratory syndrome coronavirus 2 (SARS-CoV-2), more than 700 million cases and 6 million deaths have been reported worldwide. During a global pandemic, two SARS-CoV-2 vaccines received emergency use authorization from the United States (U.S.) Food and Drug Administration, and clinical trials performed in the general population estimated the efficacy of these vaccines to be 94 to 95% in protecting against severe forms of COVID-19 (1, 2).

Patients on hemodialysis (HD) have long been known to have a greatly increased risk of death compared with the general population (3). Patients with end-stage renal disease (ESRD) have a dysregulated immune system and significant accompanying comorbid conditions (4). Several studies have demonstrated that patients who are receiving HD have a very high risk of COVID-19-related mortality (3, 5–11). Furthermore, they are vulnerable to COVID-19 infection because of their frequent visits to dialysis centers and contact with each other. Salerno et al. evaluated COVID-19 risk factors and mortality outcomes in the population undergoing HD using U.S. Medicare claims data (10). They reported that 60,090 (12.1%) had COVID-19, among whom 15,612 patients (26.0%) died.

Vaccine selection and schedule recommendations for patients undergoing HD are the same as those for the general populations. However, direct evidence of vaccine efficacy among HD patients is limited, primarily to observational data, which confirmed the benefits of vaccination (12–16). Sibbel et al. showed that vaccination with mRNA-1273 or BNT162b2 led to a lower risk of COVID-19 diagnosis and a lower risk of hospitalization or death among those diagnosed with the infection (13). Anand et al. evaluated seroconversion rates among patients undergoing HD and reported that the humoral response to SARS-CoV-2 vaccination decreases and is associated with the risk of breakthrough infection (12). Seroconversion generally occurs in most dialysis patients, but rates may be lower compared with the general population.

Most SARS-CoV-2 vaccine trials did not include patients undergoing HD. Large-scale HD cohort studies have been conducted in West (13, 16). Therefore, our objective was to estimate the effectiveness of the SARS-CoV-2 vaccine on the risk of COVID-19 infection, severe COVID-19, and mortality in patients undergoing HD using data from the National Health Insurance Service in South Korea.

## Methods

### Study design and data source

This is a retrospective observational study based on the National Health Insurance System (NHIS)-COVID-19 cohort database in South Korea. The NHIS is a single-payer system and covers more than 98% of the Korean population. It offers nationwide medical information for researchers, including sociodemographic characteristics in inpatient and outpatient records. The Korea Centers for Disease Control and Prevention (KCDC) provided data on patients diagnosed with COVID-19, including vaccination information, dates, series, and types. They confirmed the information for the COVID-19 patient, including the date of diagnosis. NHIS and KCDC generated a cohort of COVID-19-related registers merged with detailed medical information for scientific research. Physicians in South Korea are required to report COVID-19 infections. Information about COVID-19 patients before October 8, 2020, was not included because of the low probability of identification of each patient, who comprised <0.001% of the country’s population.

### Study population

**Figure 1:**
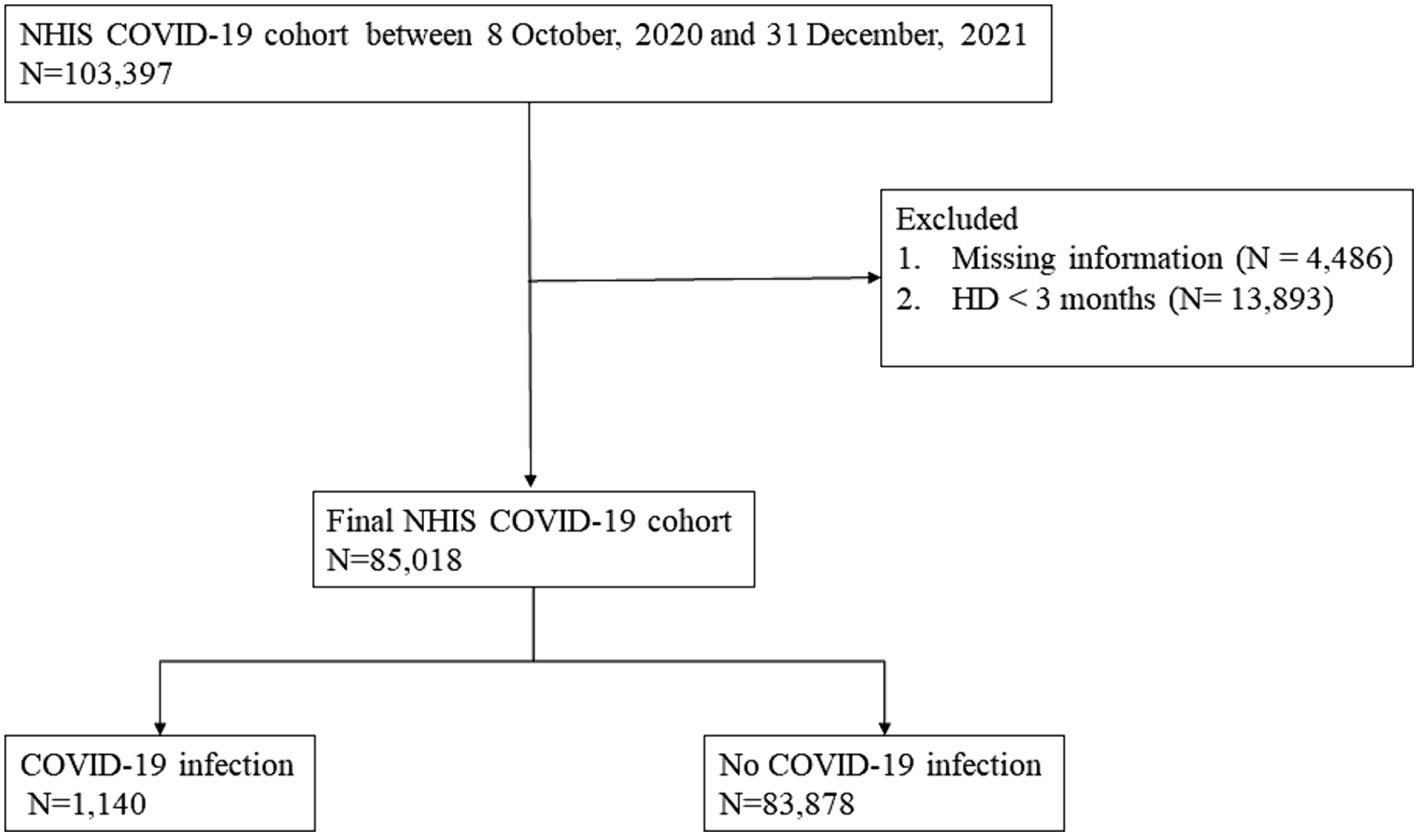
Flow chart of the study population.

The Korean NHIS has a special exemption code to provide financial support to patients who start HD. We identified adult patients (≥ 18 years) diagnosed with ESRD who underwent HD based on the presence of International Classification of Diseases (ICD) codes (N18.0–18.6, 18.9), the exemption code V001, and HD treatment codes O7020, O7021 between October 9, 2020, and December 31, 2021 (see Figure 1). Among these patients, we excluded those who had undergone HD for less than 3 months and had missing information. Patients were observed until January 31, 2022. The study was conducted according to the *Declaration of Helsinki*. The database was fully anonymized, and the requirement of informed consent was waived by the Ethics Committee of the Institutional Review Board of Hallym University Kangnam Sacred Heart Hospital (IRB No. HKS 2022–07-022).

### Study outcomes

The date of initial HD, or October 8, 2020, was defined as the index date, and patients were followed until COVID-19 diagnosis or the study end date (January 31, 2022). The primary outcome was a first positive diagnosis of COVID-19 infection based on reverse transcriptase–polymerase chain reaction assays in the database. Secondary outcomes were all-cause mortality within 28 days after the first diagnosis of COVID-19 and severe clinical events after COVID-19-related hospitalization, including administration of oxygen supply with a high-flow nasal cannula (HFNC), mechanical ventilation (MV), and continuous renal replacement therapy (CRRT).

### Covariates and vaccination status

Data included demographic characteristics (age and sex), place of residence (metropolitan and non-metropolitan areas), Medicaid beneficiary status, vaccination status, and comorbidities. Full COVID-19 vaccination status was defined as completing the primary series of COVID-19 vaccinations, namely BNT162b2 (Pfizer–BioNTech), mRNA-1273 (Moderna), ChAdOx1 nCoV-19 (AstraZeneca), NVX-CoV2373 (Novavax), and Ad26.COV2.S (Janssen/Johnson & Johnson) vaccine, or unvaccinated. Two doses are needed for the completion of the primary series. Additionally, a single Ad26.COV2.S vaccine was considered sufficient to complete the primary series as it was not approved for the second dose. Partial vaccination status was defined as having received one dose of the aforementioned vaccines. If the patient was diagnosed with COVID-19 within 14 days of vaccination, we considered them unvaccinated because of the short duration. Baseline comorbidities were defined as conditions diagnosed within 1 year before the index date using the following ICD-10 diagnostic codes (see Supplementary file 1).

### Statistical analysis

The sample was divided into three groups according to vaccine status. Distributions of continuous variables are presented as mean (standard deviation [SD]) and continuous variables as number (percent). The inverse probability of treatment weighting (IPTW) was used to control for differences in patient characteristics between the groups, and standardized mean differences (SMD) were used to assess the balance of covariates. We used propensity analysis to calculate propensity scores (PS), namely IPTW, to further evaluate the associations between vaccine status and COVID-19 infection risk (17). PS values were defined as the probability that a patient had received a full vaccination and were estimated based on fitting a multivariate logistic regression model, with age, sex, place of residence, Medicaid beneficiaries, and comorbid conditions as covariates. The covariate imbalance between the groups was assessed by evaluating SMDs for each covariate separately in the unadjusted and IPTW-adjusted cohorts. The risk of COVID-19 infection was analyzed using logistic regression analyses with IPTW, among fully and partially vaccinated, and unvaccinated patients, adjusted for age, sex, place of residence, Medicaid beneficiary status, vaccination status, and comorbid conditions (diabetes, hypertension, ischemic heart disease, heart failure, lung disease, liver disease, dementia, stroke, and cancer).

Next, we performed logistic regression analyses to assess mortality risk and COVID-19-related outcomes (use of HFNC, MV and CRRT) by vaccine status among patients infected with SARS-CoV-2. Survival curves for the three groups were estimated using the Kaplan–Meier method, and the difference was analyzed using log-rank test. We applied the Bonferroni correction for multiple comparisons and *p* < 0.0167 was considered statistically significant. Statistical analyses were performed using R version 4.0.5 (R Foundation for Statistical Computing, Vienna, Austria).^1^

## Results

### Baseline characteristics by vaccination status

Of the 85,018 patients with ESRD, 69,601 (81.9%) were fully vaccinated. The patient’s mean age was 65, and 59.5% were men. Considering all patients, 21.1% were Medicaid beneficiaries, 48% lived in metropolitan areas, 72% had hypertension, and 59.1% had diabetes. Fully vaccinated patients were younger and had a lower proportion of stroke. Differences between fully vaccinated and unvaccinated groups were reduced after weighting (see Table 1).

**Table 1:** Baseline characteristics by vaccination status.

	Unweighted				Weighted			
	Fully vaccinated (*n* = 69,601)	Partially vaccinated (*n* = 2,213)	Unvaccinated (*n* = 13,204)	SMD	Fully vaccinated (*n* = 84,964)	Partially vaccinated (*n* = 85,159)	Unvaccinated (*n* = 86,699)	SMD
Age, year	63.83 (13.01)	67.20 (13.32)	68.66 (14.16)	0.239	64.62 (12.97)	64.44 (13.99)	63.14 (15.95)	0.067
Sex				0.062				0.004
Men	41,985 (60.3)	953 (43.1)	5,845 (44.3)		34,382 (40.5)	3,4,382 (40.4)	34,824 (40.2)	
Women	27,646 (39.7)	1,260 (56.9)	7,359 (55.7)		50,582 (59.5)	50,776 (59.6)	51,875(59.8)	
Medicaid beneficiary	14,600 (21.0)	522 (23.6)	2,821 (21.4)	0.042	17,399 (20.5)	21,373 (25.1)	21,045 (24.3)	0.074
Residence				0.063				0.066
metropolitan	34,012 (48.9)	1,006 (45.5)	5,829 (44.1)		41,534 (48.9)	38,446 (45.1)	38,097 (43.9)	
Non-metropolitan	35,589 (51.1)	1,207 (54.5)	7,375 (55.9)		43,430 (51.1)	46,713 (54.9)	48,602 (56.1)	
Comorbid conditions								
Diabetes	40,870 (58.7)	1,345 (60.8)	8,019 (60.7)	0.028	50,080 (58.9)	50,870 (59.7)	50,537 (58.3)	0.02
Hypertension	50,298 (72.3)	1,584 (71.6)	9,326 (70.6)	0.024	61,430 (72.3)	60,612 (71.2)	60,520 (69.8)	0.037
Heart failure	6,953(10.0)	2,59 (11.7)	1,805 (13.7)	0.076	8,576 (10.1)	9,853 (11.6)	11,078 (12.8)	0.056
Ischemic heart disease	13,484 (19.4)	524 (23.7)	2,911 (22.0)	0.070	16,656 (19.6)	19,697 (23.1)	17,826 (20.6)	0.057
Stroke	2,699 (3.9)	138 (6.2)	997 (7.6)	0.106	3,817 (4.5)	3,827 (4.5)	3,751 (4.3)	0.005
COPD	3,666 (5.3)	155 (7.0)	978 (7.4)	0.059	4,548 (5.4)	5,676 (6.7)	5,703 (6.6)	0.037
Liver disease	4,631 (6.7)	142 (6.4)	805 (6.1)	0.015	5,598 (6.6)	5,746 (6.7)	5,601 (6.5)	0.008
Cancer	1,644 (2.4)	75 (3.4)	529 (4.0)	0.063	2,036 (2.4)	2,776 (3.3)	3,171 (3.7)	0.049

Data are number (percentage) and mean (standard deviation).COPD, chronic obstructive lung disease; SMD, standardized mean difference.

Risk of COVID-19 infection and post-diagnosis death according to vaccination status.

Of the 85,018 patients with ESRD undergoing HD, 1,140 (1.3%) were diagnosed with COVID-19 during the study period. The difference between infected and noninfected patients with COVID-19 is shown in Supplementary file 2. COVID-19 infected group had lower proportion of fully vaccinated patients (47.6% vs. 82.3%) and higher proportion of living in metropolitan area (70.5% vs. 47.7%). Table 2 compares the clinical characteristics between fully (543 [47.6%]), partially (20 [1.8%]) vaccinated and unvaccinated (577 [50.6%]) COVID-19-infected patients undergoing HD. The former are older than the unvaccinated ones, but there are no statistically significant differences between the groups for most clinical characteristics.

**Table 2:** Comparison of characteristic in COVID-19 infected patients.

	Fully vaccinated (*n* = 543)	Partially vaccinated (*n* = 20)	Unvaccinated (*n* = 577)	*p*-value
Age, year	65.74 (11.9)	66.35 (9.76)	64.17 (14.16)	0.117
Sex				
Women	212 (39.0)	12 (60.0)	238 (41.2)	0.152
Men	331 (61.0)	8 (40.0)	339 (58.8)	
Medicaid beneficiary	125 (23.0)	8 (40.0)	146 (25.3)	0.179
Residence				0.562
metropolitan	382 (70.3)	12 (60.0)	410 (71.1)	
Non-metropolitan	161 (29.7)	8 (40.0)	167 (28.9)	
Comorbid conditions				
Diabetes	331 (61.0)	16 (80.0)	337 (58.4)	0.125
Hypertension	384 (70.7)	17 (85.0)	407 (70.5)	0.373
Heart failure	54 (9.9)	3 (15.0)	54 (9.4)	0.687
Ischemic heart disease	125 (23.0)	7 (35.0)	116 (20.1)	0.174
Stroke	21 (3.9)	1 (5.0)	30 (5.2)	0.563
COPD	26 (4.8)	1 (5.0)	34 (5.9)	0.712
Liver disease	34 (6.3)	2 (10.0)	38 (6.6)	0.794
Cancer	20 (3.7)	1 (5.0)	13 (2.3)	0.322

COPD, chronic obstructive lung disease.

**Figure 2:**
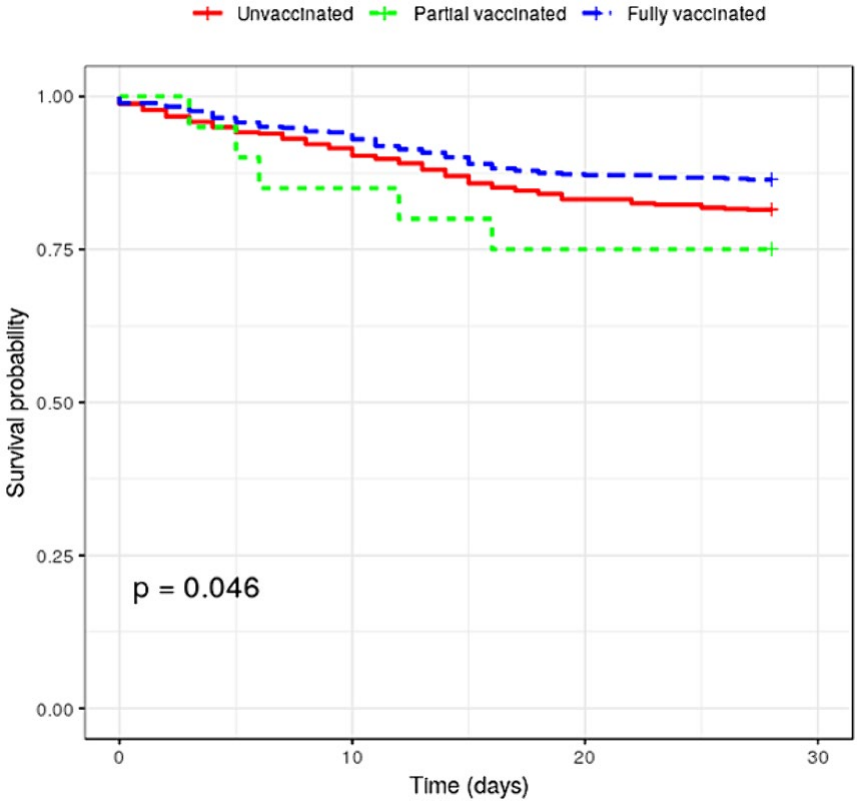
Survival curves by vaccination status.

Table 3 shows the COVID-19 rates and all-cause mortality in COVID-19 infection. Five hundred forty-three cases occurred among those fully vaccinated. During the entire study period, COVID-19 case rates were 0.018 and 0.022 per 1,000 person-days for fully and partially vaccinated patients, respectively. The adjusted risk of COVID-19 infection was significantly lower in fully [aOR (weighted), 0.147; 95% CI, 0.135–0.159] and partially [aOR (weighted), 0.172; 95% CI, 0.159–0.186] vaccinated patients compared with unvaccinated patients. One hundred eighty-six deaths (16.3%) occurred among patients with COVID-19, with 74 and 107 deaths among fully vaccinated and unvaccinated patients, respectively (see Table 3). The mortality rate after COVID-19 infection was 5.33 and 7.50 per 1,000 person-days, respectively. Mortality risk was significantly lower in fully vaccinated patients (aOR = 0.940; 95% CI = 0.901–0.0.980). The survival curves between fully vaccinated and unvaccinated patients were different (log-rank *p* < 0.001; see Figure 2).

**Table 3:** Risk of COVID-19 infection and mortality by vaccine status.

	Covid-19 diagnosis		Odds ratio (95% CI) (weighted)	
	Number	Rate per 1,000 person-days	Unadjusted	Adjusted
Unvaccinated	577	0.106	reference	reference
Partially vaccinated	20	0.022	0.176 (0.163–0.190)^*^	0.172 (0.159–0.186)^*^
Fully vaccinated	543	0.018	0.154 (0.142–0.167)^*^	0.147 (0.135–0.159)^*^
	Post-diagnosis death		Odds ratio (95% CI)	
Unvaccinated	107	7.50	reference	reference
Partially vaccinated	5	10.82	1.464 (0.468 ~ 3.872)	1.027 (0.876 ~ 1.204)
Fully vaccinated	74	5.33	0.693 (0.501 ~ 0.955)	0.940 (0.901 ~ 0.980) ^*^

^*^p < 0.0167.

### Severe clinical events among COVID-19 infected patients

**Figure 3:**
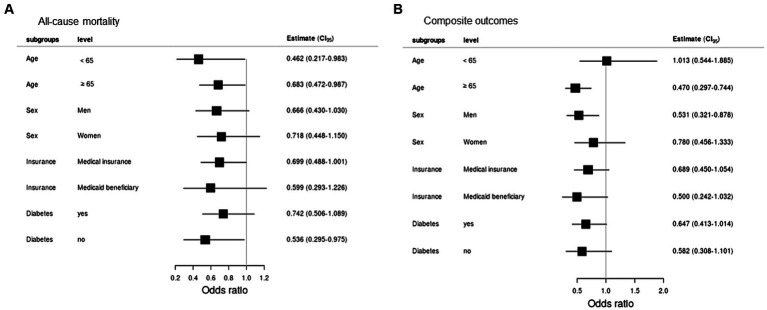
Subgroup analyses of **(A)** all-cause mortality and **(B)** composite outcomes.

The composite result occurred in 86 unvaccinated and 53 fully vaccinated patients (Table 4). Fully vaccinated patients had a lower risk of composite severe COVID-19 (aOR = 0.952; 95% CI = 0.916–0.988). The risk of application of MV (aOR = 0.972; 95% CI = 0.951–0.995) and CRRT (aOR = 0.960; 95% CI = 0.936–0.984) was higher in the unvaccinated group. All-cause mortality and composite outcomes between fully vaccinated and unvaccinated patients in subgroups showed in Figure 3.

**Table 4:** Severe clinical events among COVID-19 infected patients by vaccination status.

Outcomes		Events after COVID-19 diagnosis	Odds ratio (95% CI)	
			Unadjusted	Adjusted
Composite outcomes	Fully vaccinated	53	0.639 (0.440 ~ 0.922)	0.952 (0.916 ~ 0.988)^*^
	Partial vaccinated	4	1.509 (0.424 ~ 4.236)	1.040 (0.900 ~ 1.201)
	Unvaccinated	86	ref	ref
MV	Fully vaccinated	13	0.447 (0.223 ~ 0.848)	0.972 (0.951 ~ 0.995)^*^
	Partial vaccinated	2	2.026 (0.313 ~ 7.472)	1.040 (0.954 ~ 1.134)
	Unvaccinated	30	ref	ref
HFNC	Fully vaccinated	43	0.800 (0.526 ~ 1.210)	0.979 (0.947 ~ 1.013)
	Partial vaccinated	4	2.326 (0.649 ~ 6.604)	1.097 (0.966 ~ 1.245)
	Unvaccinated	56	ref	ref
CRRT	Fully vaccinated	15	0.403 (0.213 ~ 0.726)^*^	0.960 (0.936 ~ 0.984)^*^
	Partial vaccinated	2	1.576 (0.245 ~ 5.740)	1.023 (0.930 ~ 1.125)
	Unvaccinated	39	ref	ref

MV, mechanical ventilation; HFNC, high-flow nasal cannula; CRRT, continuous renal replacement therapy.^*^p < 0.0167.

## Discussion

This study investigated the effects of the vaccine status on the risk of COVID-19 and clinical outcomes in patients undergoing HD between October 2020 and December 2021 using data from the KCDC-NHIS COVID-19 cohort. We found that SARS-CoV-2 vaccines were effective with a lower risk of COVID-19 infection and post-diagnosis mortality in Korean patients undergoing HD who have been fully vaccinated. Furthermore, the risk of severe clinical events during hospitalization after COVID-19 infection was lower in the fully vaccinated group. Our results showed that the fully vaccinated group was younger than the unvaccinated group. The contradiction between this age distribution and vaccination status compared with the general public can be explained by the fact that very older adult patients undergoing HD who had underlying diseases or were not in good health may not have been vaccinated (18). This is because there has been controversy about the vaccine’s safety. For this reason, the analysis was performed applying weighting according to the status of the vaccine.

Herein, only 1.3% of patients undergoing HD were diagnosed with COVID-19 during the study period and the incidence was low compared with data reported by other countries. In the Registry of the European Renal Association - European Dialysis and Transplant Association, 2.9% of prevalent dialysis patients were affected by COVID-19 (9). The French national population cohort data showed a similar incidence of 3.3% among 48,669 patients undergoing HD (11). Meanwhile, the incidence of COVID-19 among U.S. Medicare patients undergoing HD has been reported at 12% (10). The rate of COVID-19 infection can differ depending on the disparity in structural differences in access to health care and the system. Home HD has been associated with a lower probability of infection (10). However, in South Korea most patients receive HD in dialysis centers. Although there is no home HD system, a joint committee was established with nephrologists and government authorities to conduct collaborative activities for patients undergoing HD during the COVID-19 outbreak in February 2020. They developed COVID-19 clinical practice guidelines for patients undergoing HD to prevent transmission and minimize the spread of COVID-19 (19). The guideline included pre-emptive activities, including early detection with rapid testing, cohort isolation, collaboration between institutions, and continuous infection monitoring. This can contribute to a low incidence rate of COVID-19 in South Korea.

In this study. The 28-day mortality rate after COVID-19 was 16.3% among the 1,140 patients undergoing HD. Although it seems difficult to directly compare the death rates of COVID-19 in various countries, our mortality rate was comparable to the reported rate in other population-based data (9–11). Other single-center data in New York reported high case mortality rates, but a selection bias for the study population, including severely ill hospitalized patients, could explain these cases (3). Although there are no Korean data on death rates by age during the study period, it appears that the death rate among Korean patients undergoing HD is more than twice that of a population-based study in Italy, where the case-fatality rate was 3.5% in the 60–69-year-old group and 12.8% in the 70–79-year-old group (20). Patients undergoing HD have long been known to have a greatly increased risk of death compared with the general population. Comorbidities may have played a significant role in explaining this substantial mortality. Herein, 59.1% had diabetes and 19.9% had a history of ischemic heart disease in this study. Compared with the unvaccinated group, the partially vaccinated group had no effect on reducing the mortality rate and severe clinical events after COVID-19 infection. There are limitations to interpretation due to the large difference in the number of patients between the two groups, but it is likely that the partially vaccinated group was unable to complete the vaccine due to poor clinical condition.

This study showed that vaccination was associated with an 85% lower relative risk of COVID-19 infection. These estimates are comparable to previous studies targeting ESRD patients, but lower than the efficacy estimated from clinical trials in the general population (1, 2, 13, 16). The mortality and severe COVID-19 event rates were higher in the unvaccinated group among patients diagnosed with COVID-19 than in the fully vaccinated group. Our estimates align with the reported real-world effectiveness estimates from several large studies of the general population (21–23). This study confirms that despite the low seroconversion rates observed in previous studies, vaccination protects against symptomatic infection and death secondary to COVID-19 in these populations. Recent studies have shown that SARS-CoV-2 vaccination induces an adequate antibody response and can be safely administered to patients undergoing HD (24–26). In the study by Moscara et al., administering the anti-SARS-CoV-2 vaccine simultaneous with other vaccinations did not affect vaccine safety or increase the risk of SARS-CoV-2 breakthrough infection (24). Nevertheless, some vaccination hesitancy remains, and some countries have insufficient uptakes of booster vaccines (27). Health-care workers play an important role in promoting vaccination in their communities. They may need strategies to provide accurate, useful vaccine information to increase vaccination rates (28).

This study included data from the entire national population with ESRD that who undergo HD. However, there are limitations associated with the study. First, observational cohort studies are vulnerable to residual confounders and biases. Although weighting is applied between the vaccinated and unvaccinated groups, it is possible that differences in the COVID-19 case rates could have confounded the results. Second, underreporting of COVID-19 cases may have led to an overestimation of mortality. This could be especially true for patients treated at home, asymptomatic patients, or those undergoing sudden death. However, most patients with symptoms were admitted to a hospital during the study period in South Korea. Third, we were unable to include cases during the Omicron variant surge. These data were unavailable because of delays in the processing of NHIS inpatients and outpatient claims. Further studies that include the Omicron variant period are planned.

In conclusion, our results demonstrate that SARS-CoV-2 vaccines are associated with a lower risk of COVID-19 infection and post-diagnosis death among patients undergoing HD. Furthermore, fully vaccinated patients had a lower risk of severe COVID-19-related complications, including administration of HFNC, MV, or CRRT. Our results support the use of COVID-19 vaccines in patients undergoing HD. However, further studies are needed to assess vaccine effectiveness on clinical outcomes during the Omicron variant period and beyond.

## Data availability statement

The raw data supporting the conclusions of this article will be made available by the authors, without undue reservation.

## Ethics statement

The studies involving humans were approved by the Ethics Committee of the Institutional Review Board of Hallym University Kangnam Sacred Heart Hospital (IRB No. HKS 2022–07-022). The studies were conducted in accordance with the local legislation and institutional requirements. Written informed consent for participation was not required from the participants or the participants’ legal guardians/next of kin because the database was fully anonymized.

## Author contributions

AC: Writing – original draft, Writing – review & editing. Y-KL: Writing – review & editing. SJ: Formal analysis, Writing – original draft. HP: Writing – original draft. DK: Writing – original draft. KY: Writing – original draft. HY: Writing – original draft. YK: Writing – original draft.

## Footnotes

^1^ https://www.R-project.org/
