# Supplementary material for: SARS-CoV-2 vaccine effectiveness and clinical outcomes in hemodialysis patients: the NHIS-COVID-19 cohort study in South Korea
Source: Front Public Health. 2024 May 9;12:1372525. doi: 10.3389/fpubh.2024.1372525 (PMC11111925; doi:10.3389/fpubh.2024.1372525)
Supplement: Supplementary file 1 [file Data_Sheet_1.PDF]

Supplementary File 1. The ICD-10 codes used by comorbidity

- Diabetes: E10-E14
- Hypertension: I10-I13, I15
- Heart failure: I50
- Ischemic heart disease: I20-I25
- Stroke: I60-64
- Chronic pulmonary disease: J42-J47, J60-67, J70.1, J70.3
- Liver disease: B18. B18, K70.4, K71.1, K71.3, K71.4, K71.5, K73, Z94.4, K70.3, K71.7, K72.1, K72.9, K74.3-6, I85, I86.4, I98.2
- Cancer: C00-C97

Supplementary File 2. Baseline characteristic by COVID-19 infection

	COVID-19 (n=1,140)	No COVID-19 (n=83,878)	SMD
Age, year	64.96 (13.1)	64.66 (13.3)	0.022
Sex			
Women	462 (40.5)	33,952 (40.5)	0.001
Men	678 (59.5)	49,926 (59.5)	
Medicaid beneficiary	279 (24.5)	17,664 (21.1)	0.081
Residence			0.477
metropolitan	804 (70.5)	40,043 (47.7)	
Non-metropolitan	336 (29.5)	43,835 (52.3)	
Fully vaccinated	543 (47.6)	69,058 (82.3)	0.781
Comorbid conditions			
Diabetes	684 (60.0)	49,550 (59.1)	0.019
Hypertension	808 (70.9)	60,400 (72.0)	0.025
Heart failure	111 (9.7)	8,906 (10.6)	0.029
Ischemic heart disease	248 (21.8)	16,671 (19.9)	0.046
Stroke	52 (4.6)	3,782 (4.5)	0.003
COPD	61 (5.4)	4,738 (5.6)	0.013
Liver disease	74 (6.5)	5,504 (6.6)	0.003
Cancer	34 (3.0)	2,214 (2.6)	0.021
